# Development of Cell-Carrying Magnetic Microrobots with Bioactive Nanostructured Titanate Surface for Enhanced Cell Adhesion

**DOI:** 10.3390/mi12121572

**Published:** 2021-12-17

**Authors:** Junyang Li, Lei Fan, Yanfang Li, Tanyong Wei, Cheng Wang, Feng Li, Hua Tian, Dong Sun

**Affiliations:** 1 Department of Biomedical Engineering, City University of Hong Kong, 83 Tat Chee Avenue, Hong Kong, China; junyangli3-c@my.cityu.edu.hk (J.L.); leifan-c@my.cityu.edu.hk (L.F.); yanfangli2-c@my.cityu.edu.hk (Y.L.); tanyong.wei@my.cityu.edu.hk (T.W.); 2 Centre for Robotics and Automation, Shenzhen Research Institute of City University of Hong Kong, Shenzhen 518057, China; 3 Hong Kong Center for Cerebro-Cardiovascular Health Engineering (COCHE), Science Park, Hong Kong, China; 4 Department of Orthopaedics/Engineering Research Center of Bone and Joint Precision Medicine/Beijing Key Laboratory of Spinal Disease Research, Peking University Third Hospital, 49 North Garden Road, Haidian District, Beijing 100191, China; wangchengbjmu@bjmu.edu.cn (C.W.); ifmed@sina.com (F.L.); tianhua@bjmu.edu.cn (H.T.)

**Keywords:** magnetic microrobots, nanostructured titanate surface, cell carrying, enhanced cell adhesion

## Abstract

Cell-carrying magnet-driven microrobots are easily affected by blood flow or body fluids during transportation in the body, and thus cells often fall off from the microrobots. To reduce the loss of loaded cells, we developed a microrobot with a bioactive nanostructured titanate surface (NTS), which enhances cell adhesion. The microrobot was fabricated using 3D laser lithography and coated with nickel for magnetic actuation. Then, the microrobot was coated with titanium for the external generation of an NTS through reactions in NaOH solution. Enhanced cell adhesion may be attributed to the changes in the surface wettability of the microrobot and in the morphology of the loaded cells. An experiment was performed on a microfluidic chip for the simulation of blood flow environment, and result revealed that the cells adhered closely to the microrobot with NTS and were not obviously affected by flow. The cell viability and protein absorption test and alkaline phosphatase activity assay indicated that NTS can provide a regulatory means for improving cell proliferation and early osteogenic differentiation. This research provided a novel microrobotic platform that can positively influence the behaviour of cells loaded on microrobots through surface nanotopography, thereby opening up a new route for microrobot cell delivery.

## 1. Introduction

Cell-based therapy has been widely used to treat various diseases, such as brain disorder [1], knee cartilage degeneration [2], central nervous system disorders [3] and cancer [4]. Traditionally, therapeutic cells are injected directly [5,6,7,8] or encapsulated with biocompatible polymers prior to administration to targeted damage sites [9,10,11,12]. However, these methods lack precise targeting ability and may cause the opening of wounds. Therefore, magnet-driven microrobots have increasingly attracted attention for its precise positioning ability, minimal invasiveness and harmlessness to biological substances [13,14,15,16,17,18,19,20,21]. Cell delivery using magnetic microrobots with different microgeometries has been reported in vitro and in vivo [22,23,24]. A U-shaped magnetic microrobot was developed to utilize a magnetic field in transporting a single mouse embryo [25,26]. Magnetic porous cuboid and cylindrical microrobots were designed to transport human embryonic kidney (293 cells) in vitro [27]. Porous microrobots carrying mesenchymal stem cells (MSCs) were manipulated inside the joint cavity of a rabbit knee for cartilage repair [28]. Cylindrical, hexahedral and spherical microrobots carrying MSCs from a human nose were manipulated inside the intraperitoneal cavity of a nude mouse for stem cell transplantation [29]. In our previous study, a burr-like porous spherical microrobot was designed to carry and deliver cells in vivo [30]. In all the aforementioned approaches, cells were co-cultured with magnetic microrobots with various microgeometries and injected into blood vessels, intraperitoneal cavities or joint cavities. However, a microrobot injected into the human body is affected by the blood stream or other body fluids, and cells can easily fall off from the microrobot before it reaches a targeted part of the body. Thus, enhancing the cell adhesion ability of microrobots and further reducing cell loss during transportation are challenges that should be urgently addressed.

Cell behaviour can be affected by substrate nanotopography, such as titanate or titania (TiO_2_) nanostructures, and their use has become an attractive strategy for regenerative medicine and advanced tissue engineering [31,32,33,34,35]. Rat bone MSCs cultured on electrospun TiO_2_ nanofiber-modified implantable resins show increased adhesion, proliferation and osteogenic differentiation capacity [36]. The adhesion and propagation of MC3T3-E1 osteoblasts with elongated filopodia substantially improve with the topography of TiO_2_ nanotubes grown through anodisation [33]. Titanate nanowires formed on titanium implants mimic the natural extracellular matrix and can promote MSC adhesion and proliferation [37]. Titanium-based surface nanotexture grown in macroscopic titanium implants or materials has many advantages. However, designing and manufacturing cell-carrying magnetic microrobots with appropriate nanoscale surface morphology have not been conducted.

This paper reports the design and fabrication of a magnetic microrobot with a bioactive nanostructured titanate surface (NTS), which can improve cell adhesions and minimise the loss of loaded cells on the microrobot. Magnetic microrobot skeletons with burr-like porous spherical structures were used, which can enhance magnetic driving capability and cell-carrying capacity according to a preliminary work [30]. The microrobot was coated with nickel to achieve magnetic actuation and titanium to produce an NTS outside the microrobot through a reaction in NaOH solution. The crystal structure and atomic composition of the NTS were studied through X-ray powder diffraction (XRD) and energy dispersive X-ray spectroscopy (EDS). The water contact angle (WCA) test results of NTS samples showed that cell adhesion can be increased by adjusting the wettability of the microrobot from hydrophobic to hydrophilic [37,38]. In addition, the morphology of the MSCs and HEK-293T cells seeded on NTS underwent considerable changes, that is, pseudopodium extension and cell elongation. These changes resulted in stable cell adhesion. The microrobot carrying HEK-293T cells was placed in a microfluidic chip that mimicked the blood flow environment to verify the enhancement of cell adhesion. The experimental results showed that cells adhered more closely to the microrobot with an NTS and were not greatly affected by the flow. The cell viability and protein absorption tests and alkaline phosphatase (ALP) activity assay showed that an NTS can positively affect the behaviour of the carried cells, such as cell proliferation, protein absorption and early osteogenic differentiation and thereby greatly benefit cell therapy.

## 2. Materials and Methods

### 2.1. Microrobot Fabrication

The designed microrobot was fabricated with Su-8 photoresist and a two-photon direct writing system (Nanoscribe GmbH, Karlsruhe, EL, Germany) and coated with 150 nm nickel and 50 nm titanium materials. Second, the microrobots were immersed in NaOH aqueous solutions with different concentrations (1, 3 and 5 mol/L) at room temperature for various reaction times (1, 6 and 12 h). The NaOH solution was prepared using NaOH pellets (Sigma Chemical Company, St. Louis, MO, USA). Third, the as-prepared robots were gently washed with ultra-pure water until the pH of the water dropped to 7 to obtain a magnetic microrobot with an NTS. Finally, the cells were seeded onto the microrobots for culturing.

### 2.2. Apparatus

The microrobot samples were coated with nickel and titanium with a Q150TS sputtering system (Quorum Technologies, Newhaven, ES, UK). The morphology of the fabricated microrobots with NTS and cells were observed through scanning electron microscopy (SEM) system (FEI, Nova 450). The crystal structures of NTS were investigated using an XRD-6100 system (Shimadzu, Nakagyo-ku, Kyoto, Japan) with Cu Kα radiation (λ = 0.15406 nm) from 5 °C to 65 °C. Tube voltage and current were set at 40 kV and 40 mA, respectively. Data were collected in 0.02 °C steps for 1 s per step. The atomic composition of the NTS was further characterised with an EDS system (Oxford Instrument, Oxford, Oxfordshire, UK). The WCA test was investigated using contact angle meter (Chengding Technologies, Dongguan, Guangdong, China). Optical density (OD) was determined using a SpectraMax M5e microplate reader (Molecular Devices, San Jose, CA, USA). The magnetic control experiment was performed using a self-constructed magnetically actuated micromanipulation system in a microfluidic chip (Figure S2 and Video S3).

### 2.3. Cell Culture on Microrobot

The MSCs were maintained in an alpha minimum essential medium (α-MEM), and HEK-293T cells were maintained in Dulbecco’s modified Eagle’s medium (DMEM) at 37 °C in a humidified atmosphere with 5% CO_2_. The cells were trypsinised and resuspended at a concentration of 1 × 10^6^ cells/mL. Subsequently, the cells were then seeded onto the microrobots and maintained in a cell culture medium for 24 h. Finally, the cell-cultured microrobot was detached for further use.

### 2.4. WCA Test

NTS wettability was characterised using static WCA measurement. First, a water droplet (2 μL) was carefully applied onto the NTS. Then, an image of the droplet was captured for WCA analysis. The contact angles were measured at three different spots for each sample, and the mean was taken as the static WCA.

### 2.5. Morphology of Cells on NTS

MSCs and HEK-293T cells were maintained in α-MEM and DMEM and then resuspended at a concentration of 1 × 10^6^ cells/mL. The two types of cells were seeded onto different specimens and maintained in cell culture medium for 1 day. Afterwards, the cell morphology of the samples was observed through SEM.

### 2.6. Cell Viability Tests

MTT assay was used in examining the viability of MSCs seeded onto glass substrates with and without NTS. After 1, 3 and 5 days of co-cultivation, each specimen was incubated with 100 µL of MTT solution (Sangon Biotech, 0.5 mg/mL) at 37 °C for 4 h for the formation of formazan crystals in viable cells. Then, dimethyl sulfoxide (Sigma Chemical Company, St.Louis, MO, USA) at 150 µL was added to dissolve the crystals. The OD of the solutions corresponded to the level of viability.

### 2.7. Protein Adsorption Assay

Fluorescein isothiocyanate-labelled albumin (FITC-albumin; Sigma Chemical Company, St.Louis, MO, USA) was used as a model protein. For the qualitative experiment, the protein solution (5 mg/mL protein in saline) at 500 μL was pipetted into the microrobots with and without NTS. After incubation for 30, 60 and 120 min at 37 °C, nonadherent proteins were removed by washing the microrobots three times with deionised water. Fluorescent intensity, which represents the adsorption ability of the microrobots, was calculated with Image J. For the quantitative experiment, protein adsorption was performed as follows: First, glass samples with and without NTS were immersed in protein solution for different time periods of 30, 60 and 120 min. Next, the residual protein content in the same volume of supernatant was quantitatively tested using a plate reader at 595 nm because protein content is proportional to absorbance at a certain range (with reference to the protocol of the Bradford protein assay kit from Beyotime Biotech, Songjiang, Shanghai, China). Finally, protein adsorption was calculated as the total protein amount minus the protein content in the supernatant.

### 2.8. Alkaline Phosphatase Activity Assay

ALP activity was qualitatively and quantitatively evaluated using commercially available kits. In the qualitative assay, MSCs at a density of 2 × 10^4^ cells were grown on glass samples with and without NTS and cultured in osteogenic differentiation media (Gibco, REF#: A10069-01). After 3 and 7 days of co-cultivation, the cells on the specimens were washed with phosphate buffer saline (PBS), fixed with 4% paraformaldehyde and stained with BCIP/NBT ALP colour development kit (Beyotime Biotech, Songjiang, Shanghai, China).

In the quantitative assay, the cells cultured on the samples were rinsed with PBS and lysed in lysis buffer containing 0.1% Triton X-100 (Sigma Chemical Company, St. Louis, MO, USA). After 2 min of centrifugation, the supernatant was used in measuring ALP activity with an alkaline phosphatase assay kit according to the manufacturer’s protocol (Beyotime Biotech, Songjiang, Shanghai, China). The measurement was based on the conversion of colourless p-nitrophenyl phosphate to coloured p-nitrophenol after co-incubation for 30 min at 37 °C. The results were normalised to the total intracellular protein content determined with a bicinchoninic acid protein assay kit (Beyotime Biotech, Songjiang, Shanghai, China). The total intracellular protein content was expressed in nanomoles of produced p-nitrophenol per minute per milligram of protein (nmol/min/mg protein).

### 2.9. Cell Adhesion Ability Test

Experiments involving the adhesion of HEK-293T cells on the microrobot with an NTS were performed. HEK-293T cells were maintained in DMEM and resuspended at a concentration of 1 × 10^6^ cells/mL. After seeding onto the microrobot and maintenance in DMEM for 6 h, the cells-cultured microrobots were stained for 5 min with 100 nM MitoTracker Red 580 (Molecular Probes, Eugene, OR, USA). After the bubbles in the chip were removed, the stained HEK-293T cell-cultured microrobots were detached and injected into the inlet of the microfluidic chips. Negative pressure was supplied to the outlet of the main channel with a syringe pump (LSP01-2A, Longer Pump). The stained cells-cultured microrobots were separately flushed for 1 min at three different flow rates of 50, 100, and 250 µL/min. Fluorescence photographs were obtained under the same confocal level, exposure time and excitation wavelength, among other conditions, to track the decrease in fluorescence area and demonstrate the degree of cell dropout.

## 3. Results

### 3.1. Characterizations

**Figure 1:**
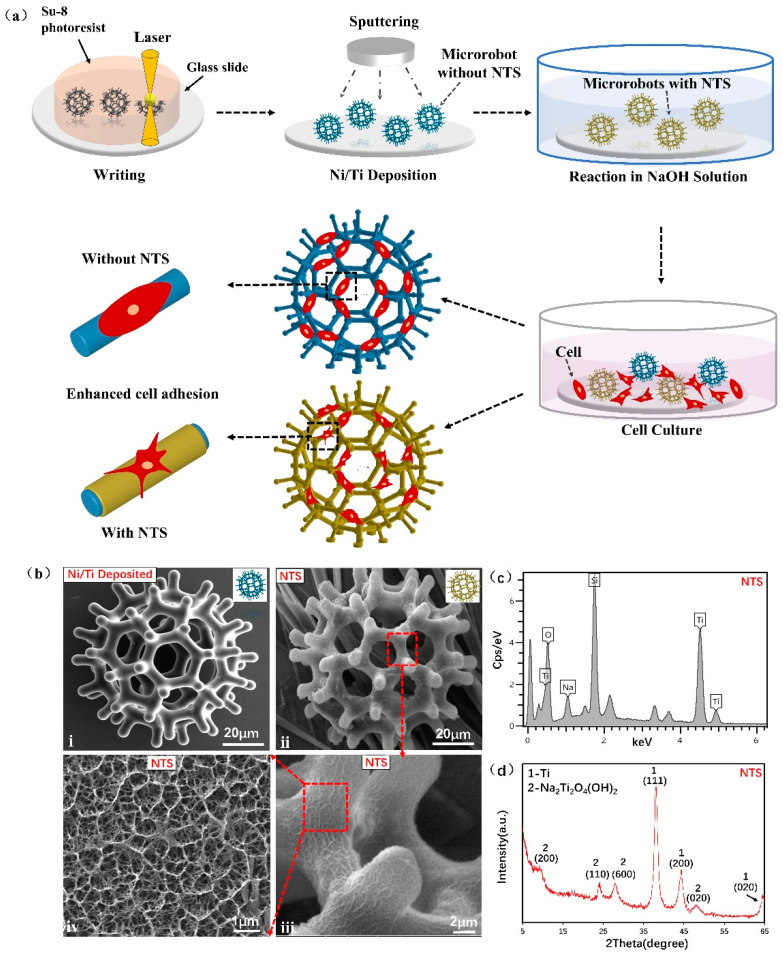
Preparation of magnetic microrobots with NTS: (**a**) Fabrication procedures of the microrobots with NTS including writing, Ni/Ti deposition, chemical reaction in NaOH solution and cell culture process; (**b**) SEM images of the microrobots with NTS: (i) Microrobot deposited with Ni for 150 nm and Ti for 50 nm (without NTS); (ii) Microrobot with NTS modified with 5 mol/L NaOH for 6 h; (iii,iv) High-magnification SEM pictures of NTS outside the microrobot; (**c**) EDS spectrum of NTS sample. (**d**) XRD pattern of NTS sample.

The effectiveness of a burr-like porous spherical structure in enhancing magnetic driving ability and cell-carrying capabilities has been demonstrated [30]. Therefore, the same microrobot structure (diameter including burrs: 90 μm) was used in this study. Figure 1 illustrates the microrobot fabrication procedures: writing, nickel/titanium deposition, chemical reaction in NaOH solution and cell culture. Compared with the microrobot without NTS, the microrobot with NTS exhibited considerably enhanced cell adhesion ability.

Figure 1b(i) shows the SEM image of the microrobot where nickel and titanium layers were successfully deposited. The outermost layer of the microrobot was sputtered with nickel layer for magnetic actuation and titanium layer as titanium source for NTS generation. Figure 1b(ii) illustrate the SEM images of the microrobot treated in NaOH to form a sodium titanate nanostructure on the outermost titanium surface. The red dotted frames in Figure 1b(iii,iv) are the high-magnification images of the NTS. The images showed the well-aligned titanate nanostructure that grew upwards from the NaOH solution and had a diameter of hundreds of nanometres and length of several micrometres. This nanostructured titanate layer was tightly connected to the titanium layer, and no exfoliation or crack appeared. These effects might have contributed to in situ construction at room temperature and formation of a stress-releasable porous nanostructure [38].

The atomic composition of the NTS sample was characterised through EDS. The sampling point was taken from the surface of the sample. The spectrum in Figure 1c confirmed that the NTS sample prepared through chemical reaction in NaOH solution contained sodium, titanium, oxygen and silicon (excluding impurity elements). Silicon was detected in the sample because it is the basic component of the glass substrate. To determine the crystal structure, we measured the XRD pattern of the NTS sample (Figure 1d). After the diffraction peaks of residual titanium were removed, the diffraction peaks corresponded to (200), (110), (600) and (020) lattice planes. Therefore, the structure of the NTS sample was indexed as Na_2_Ti_2_O_4_(OH)_2_, which has a body-centred orthogonal structure (ICDD; File No. 00-057-0123). In this reaction, the sputtered titanium layer of the microrobot acted as a biocompatible material and titanium source. The reaction to form the surface of an NTS in the chemical process is represented by the following equation:

2Ti + 2NaOH + 4H_2_O → Na_2_Ti_2_O_4_(OH)_2_ + 4H_2_↑

### 3.2. Surface Wettability

**Figure 2:**
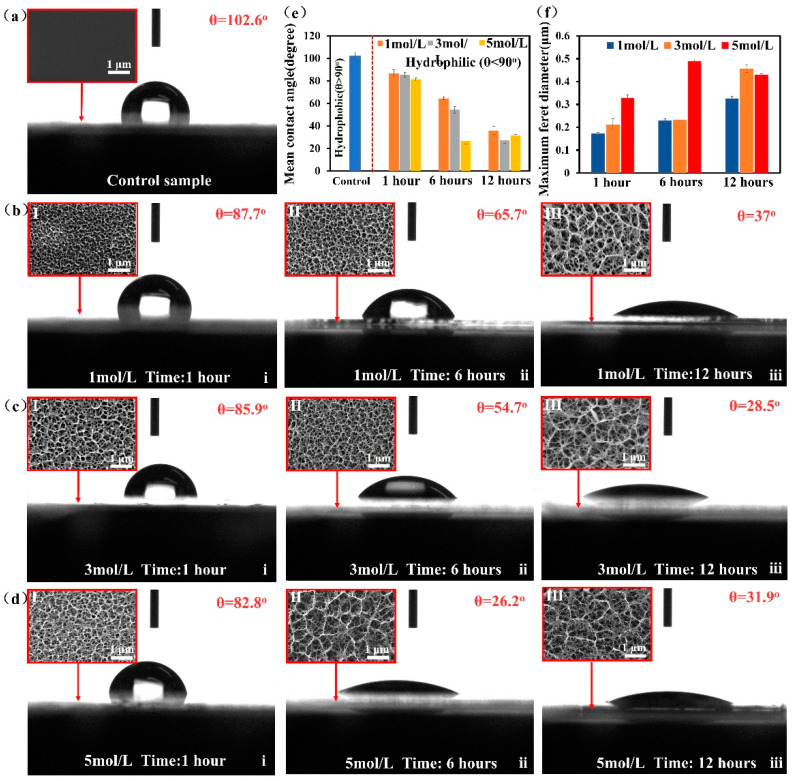
Surface wettability of the Ni/Ti samples: (**a**) WCA and SEM images of control sample; (**b**) WCA and SEM images of NTS modified with 1 mol/L of NaOH for 1, 6 and 12 h; (**c**) WCA and SEM images of NTS modified with 3 mol/L of NaOH for 1, 6 and 12 h; (**d**) WCA and SEM images of NTS modified with 5 mol/L NaOH for 1, 6 and 12 h; (**e**) Mean WCA vs. reaction time with various NaOH concentrations (n = 3); (**f**) Mean ferret diameter vs. reaction time with various NaOH concentrations (n = 3). Error bars indicate SD.

Surface wettability is an important property of biomaterials. Hydrophilic surfaces usually have better cell adhesion than hydrophobic surfaces [39,40,41]. After treatment with NaOH solution (1–5 mol/L) for 1–12 h at room temperature, a surface wettability test was performed on the Ti/Ni-coated square glass. The untreated titanium/nickel coated glass was used as the control (Figure 2a). The WCA of the control sample was approximately 102.6 °C, whereas the minimum WCA of the treated sample was approximately 26.2 °C (Figure 2d(ii)). After the nanostructures were generated, WCA decreased significantly with increasing NaOH concentration and reaction time, indicating that surface wettability can be adjusted from hydrophobic to hydrophilic by the NTS outside the titanium/nickel sample (Figure 2e).

This improvement can be attributed to the chemical structure of Na_2_Ti_2_O_4_(OH)_2_. The hydroxyl groups in NTS easily absorb water from the environment. Another possible factor is the nano-characteristics of titanates. The structural evolution of the NTS sample was characterised through SEM. The maximum ferret diameter (the longest point-to-point distance from the periphery of the area) was used to represent the irregular shape of the NTS pore. Compared with the smooth surface in Figure 2a, increase in NaOH concentration and reaction time resulted in the formation of a long nanonetwork and large pores (Figure 2b–d inset, red frame). The corresponding histograms in Figure 2e,f show that WCA decreased as the diameter of the sample hole increased. Figure 2b–d show that the hydrophilicity and ferret diameter of the surface increased with increasing reaction time and reaction concentration. This surface revealed optimal hydrophilicity and the largest ferret diameter when the reactant concentration was 5 mol/L and the reaction time was 6 h. Therefore, we chose these two parameters as the optimal conditions to treat microrobots to produce NTS on their surface. The microrobots were then compared with the microrobot without NTS in subsequent experiments. In summary, in this work, the surface morphology of the microrobot with titanium/nickel coating changed at the nanometer level after alkali treatment, which further adjusted the surface wettability and ultimately affected the adhesion of attached cells.

### 3.3. Cell Morphology Assay

**Figure 3:**
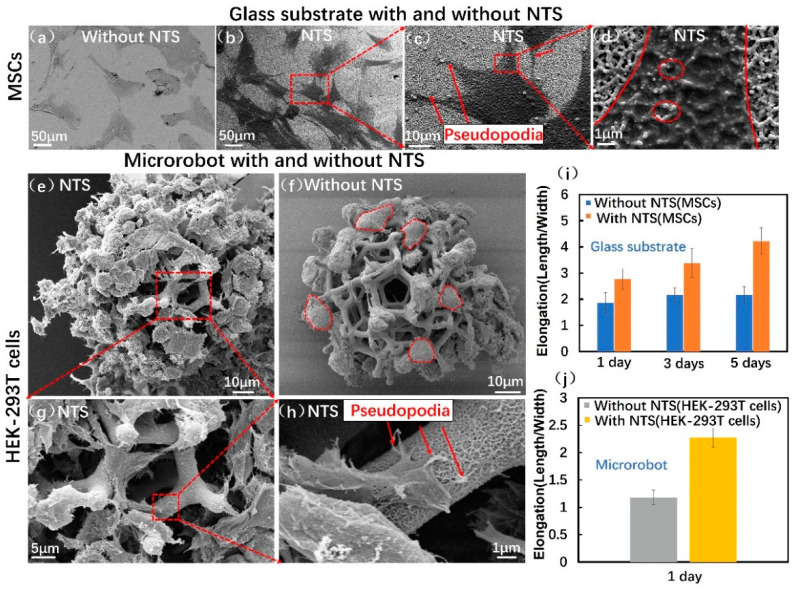
Cell morphology on NTS: (**a**) SEM image of MSCs on glass substrate without NTS after 1 day of incubation; (**b**) SEM image of MSCs on NTS sample after1 day of incubation; (**c**) SEM image of single MSC with protrusive pseudopodia on NTS sample; (**d**) High-magnification SEM image of interplay between the MSC and the NTS; (**e**) SEM image of HEK-293T cells on microrobot with NTS after 1 day of incubation; (**f**) SEM image of HEK-293T cells on microrobot without NTS; (**g**) High-magnification SEM image of HEK-293T cells on microrobot with NTS; (**h**) High-magnification SEM image of single HEK-293T cell with protrusive pseudopodia on microrobot with NTS; (**i**) Histogram representing the average elongation of MSCs on glass substrate with and without NTS cultured for 1, 3, and 5 days (n = 100); (**j**) Histogram representing the average elongation of HEK-293T cells on microrobot with and without NTS cultured for 1 day (n = 100). Error bars indicate SD.

Cell adhesion is sensitive to substrate topography [42,43,44]. The adhesion morphology of MSCs and HEK-293T cells seeded on an NTS (5 mol/L NaOH, 6 h reaction time) and control sample (Without NTS; coated with Ni/Ti layer) were examined through SEM. Figure 3a,b illustrate that the glass substrate with NTS and the control sample exhibited good cell morphology. The MSCs on the control sample (Figure 3a) were more uniform and rounded. Figure 3b,c illustrate that the MSCs attached to the NTS sample had long pseudopodia (in red arrows) that protruded and penetrated the surrounding substrates. Figure 3d shows that the protrusions of the pseudopodia stretched into the nanopores of the NTS and made close contact with the surface. This contact indicated a positive effect that enhanced cellular adhesion.

Similarly, HEK-293T cells were attached to the microrobot with NTS and control sample. This finding demonstrated cell adhesion ability. Figure 3e,g,h illustrate that the cells on the microrobots with NTS had more and longer pseudopodia and the cells on the control sample was more rounded (Figure 3f). The histogram results of cell elongation in Figure 3i,j showed that the aspect ratio of length to width of the MSCs and HEK-293T cells on the NTS sample was twice that of the control sample. This quantification of cell elongation was analysed using Image J. These typical morphology and extended pseudopodia demonstrated that the titanite nanostructures synthesised through the reaction in NaOH solution enabled cells to probe their nanoenvironments and produce stable adhesion. All these results suggested that the NTS has advantages as a bioactive surface of microrobots because it can improve cell adhesion as compared with unmodified surfaces.

### 3.4. Cell Biological Evaluation

**Figure 4:**
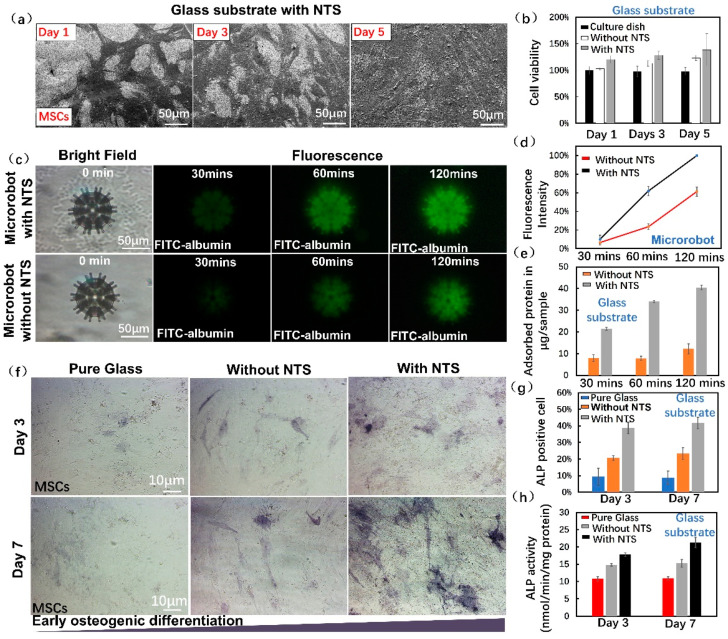
Cell biological evaluation on NTS: (**a**) SEM images of MSCs on glass substrate with NTS with days 1, 3 and 5 of culture; (**b**) Cell viability of MSCs on different substrates with days 1, 3 and 5 of culture (n = 3); (**c**) Bright field and fluorescence images of the FITC-albumin absorption on microrobot with and without NTS after 30, 60 and 120 min of incubation (n = 3); (**d**) Fluorescence intensity of microrobots with and without NTS vs. incubation time in FITC-albumin solution (n = 3); (**e**) Adsorption of protein onto glass substrate with and without NTS vs. incubation time in FITC-albumin solution (n = 3); (**f**) ALP staining images of MSCs grown on different substrates with days 3 and 7 of culture; (**g**) Percentages of ALP-positive cells on different substrates on days 3 and 7 of culture; (**h**) ALP activity of MSCs cultured on different substrates after osteogenic induction for 3 and 7 days (n = 3). Error bars indicate SD.

To investigate cell viability on the magnetic microrobots before and after the surface nanostructured treatment, 3-(4,5-dimethylthiazol-2-yl)-2,5 diphenyltetrazolium bromide (MTT) assays were performed on MSCs, which were cultured on glass substrates with NTS. Cells cultured on glasses without an NTS were used as the controls. In Figure 4a, SEM images were obtained on days 1, 3 and 5 of MSC culture on the glass substrates with NTS. The cells exhibited healthy morphology and proliferated on NTS. Figure 4b shows the absorbance of generated formazan (positively correlated with cell viability) on the surfaces of different substrates. Compared with cells in the control group, the cells in the treated group had higher cell activity after days 1, 3 and 5 of culture. This result indicated that the NTS is conducive to cell proliferation.

When the microrobots reached the target position in the body, the adsorption of the extracellular matrix adhesive proteins onto the microrobots was the first step and further affected cell differentiation through integrin binding sites [45]. FITC-albumin was selected as the model protein for the determination of the adsorption efficiency of the microrobots. As shown in Figure 4c, the microrobots with and without NTS were immersed in FITC-albumin solutions for 30, 60 and 120 min. Microrobots with NTS demonstrated the highest fluorescence intensity after 120 min of immersion. Therefore, in Figure 4d, we define this intensity as 100% fluorescence intensity (ImageJ calculation). The fluorescence intensities observed at other conditions, such as 30 and 60 min, were converted to percentages below 100%. Through this set of experiments, it can be seen that microrobots with NTS had stronger fluorescence intensities than those without NTS. Qualitative and quantitative experiments on protein adsorption in Figure S3 and Figure 4e were performed on treated glass samples. Compared with the control sample, the NTS sample adsorbed more proteins, indicating that the nanoscale surface had a higher level of adhesion protein adsorption.

ALP activity is an initial indicator of early osteogenic differentiation and osteogensis activity [46,47]. Thus, ALP staining was employed to evaluate the osteogenic differentiation of MSCs cultured on different surfaces. The dark color of the stain indicates strong ALP expression. After osteogenic induction, MSCs grown on NTS and two control samples were detected 3 and 7 days, respectively. As shown in Figure 4f,g, the percentage of ALP-positive cells on the NTS sample was significantly higher than the percentages in the pure glass and samples without NTS. Furthermore, ALP activity increased from 3 days to 7 days, especially in the cells on the NTS samples. NTS has an advantage as a bioactive surface for microrobots because it can stimulate early bone formation to higher degree than unmodified surfaces.

### 3.5. Verification of Cell Adhesion Ability in Microfluidic Chip

**Figure 5:**
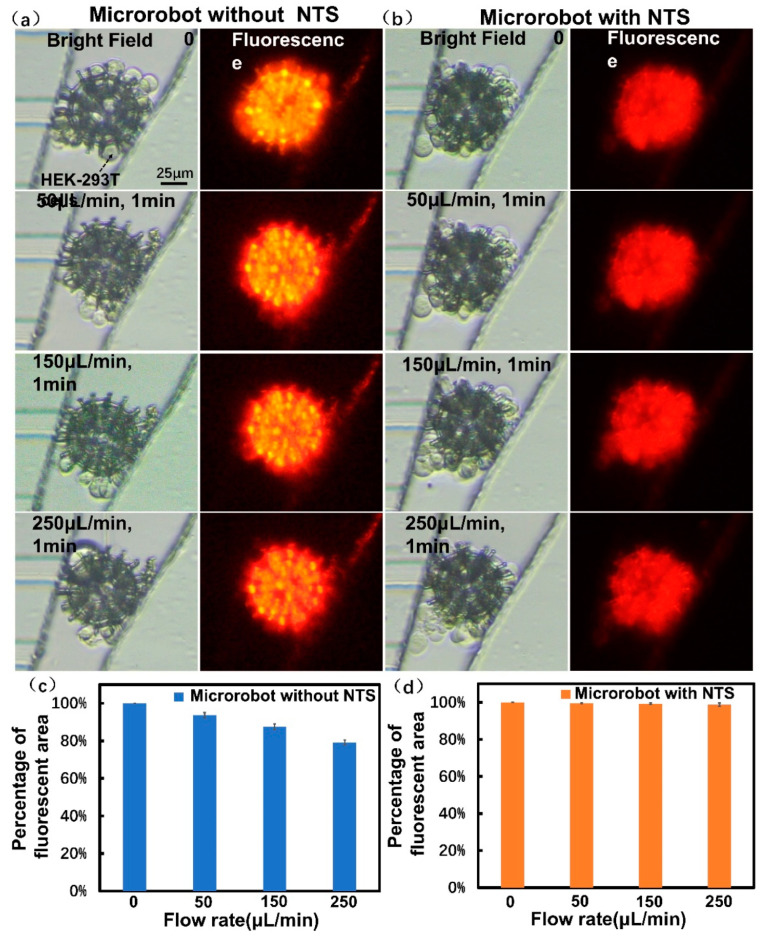
Verification of cell adhesion ability in microfluidic chip: (**a**) HEK-293T cells shed from the microrobot without NTS by passing through different flow rate; (**b**) HEK-293T cells adhered closely to the microrobot with NTS by passing through same volume flow rate; (**c**) The histogram of fluorescent area for microrobot without NTS by passing through different volume flow rate of 50 µL/min, 150 µL/min and 250 µL/min in one minute (n = 3); (**d**) The histogram of fluorescent area for microrobot with NTS by passing through different volume flow rate of 50 µL/min, 150 µL/min and 250 µL/min in one minute (n = 3). Error bars indicate SD.

Cell adhesion ability was further tested on the NTS-based microrobot loaded with HEK-293T cells. The experiments were performed in a microfluidic chip that consisted of eight blood vessel-like microchannels that mimicked the vascular network (Figure S1). The adhesion ability of the cell-loading microrobot was tested by allowing the robot to pass through flows with different volume flow rate into the microchannels. Figure 5a illustrates the cells shed from the inlet at a maximum volume flow rate of 250 µL/min. Using the volume flow rate/velocity conversion formula (details are found in the Supplementary File), the maximum flow velocity was approximately 8 cm/s in one single microchannel. This corresponding blood flow velocity was close to the vein velocity of human [48]. The microrobot without NTS after being flushed by the fluid at different volume flow rate was shown in Video S1. By contrast, no remarkable change in cells was observed when the microrobot with an NTS was used under the same condition, as shown in Figure 5b and Video S2. The fluorescent area represented the carried cells on the microrobots calculated with Image J. Figure 5c shows that the fluorescent area of the microrobot without an NTS was reduced by 20% after at different flow rates. Figure 5d shows that the fluorescent area of the microrobot with an NTS was constant at the same volume flow rates. These results revealed that the carried cells attached to the microrobots with NTS. This finding can be attributed to the fact that the shear force on the cells loaded on the microrobot increases as the flow rate increases (details are found in the Supplementary File). This finding also proves that our designed microrobot with NTS is relatively resistant to the shear force of blood flow and prevents cell loss during transportation in vivo.

## 4. Discussion

Cell-releasing ability is another important criterion to be considered in microrobot design. Our previous work showed that loaded cells can be spontaneously released from microrobots without NTS onto surrounding tissues [30]. The results in Figure 4 indicated that the microrobot with an NTS can enhance the adhesion ability of cultured cells during transportation. To determine whether an NTS affects the cell-releasing ability of the robot, we designed a cell-releasing experiment in the microfluidic chip. The 3 day incubation results confirmed that the NTS did not affect the cell-releasing ability of the designed microrobot after reaching the target site (Figure S4).

Some issues remain to be solved in future studies. First, the developed microrobots utilised biocompatible but non-degradable materials. Therefore, further optimisation of the microrobot with degradable materials is necessary for the inhibition of immune activation and thrombi formation. We designed a degradable microrobot made of synthetic composite materials [49], which can provide a reference for our future work. Second, the other surface properties of microrobots, such as surface charge, surface energy and surface oxidation, affect cell attachment and subsequent behaviour. The surface modification technology for microrobots needs to be advanced in order that more intelligent microrobots can be produced for regenerative medicine applications.

## 5. Conclusions

This paper reported the successful design and fabrication of a magnetic microrobot with an NTS for enhanced cell adhesion. The 3D laser lithography was used in fabricating the skeletons of the microrobots. This method provided sufficient flexibility for the optimisation of the microgeometry of the microrobot’s structure. The reaction in alkaline solution was used in regulating the nanoscale surface morphology of the magnetic microrobot after coating with nickel and titanium. The microrobot was fabricated and bonded to a glass substrate, and the microrobot was prevented from falling or being damaged by conducting the entire reaction process at a mild room temperature. WCA test and cell characteristics, including prolonged pseudopodia and cell elongation, demonstrated that the proposed microrobot can promote cell adhesion. The cell adhesion experiment in the microfluidic chip further confirmed that the cells tightly adhered to the designed microrobot with an NTS and were not be affected by flow shock. In addition, the results of MTT assay, protein absorption test and ALP activity assay showed that the cell proliferation and early osteogenic differentiation of NTS samples can be improved. The success of this study revealed that cell behaviour on magnet-driven microrobots can be greatly improved through the nanoscale surface functionalisation for precision medical treatments.

## Supplementary Materials

The following are available online at https://www.mdpi.com/article/10.3390/mi12121572/s1, Figure S1: Design of a microfluidic chip, Figure S2: Control of a MSCs-cultured microrobot with NTS in a microfluidic chip, Figure S3: Fluorescence images of the FITC-albumin absorption, Figure S4: Results of cells releasing ability, Video S1: Cell adhesion ability of microrobot without NTS in a microfluidic chip, Video S2: Cell adhesion ability of microrobot with NTS in a microfluidic chip, Video S3: Magnetic control experiment in a microfluidic chip.
