# Supplementary material for: Development of Cell-Carrying Magnetic Microrobots with Bioactive Nanostructured Titanate Surface for Enhanced Cell Adhesion
Source: Micromachines (Basel). 2021 Dec 17;12(12):1572. doi: 10.3390/mi12121572 (PMC8707319; doi:10.3390/mi12121572)
Supplement: Supplementary file 1 [file micromachines-12-01572-s001.zip › micromachines-1473619-supplementary.pdf]

Article

Development of Cell-carrying Magnetic Microrobots with Bioactive Nanostructured Titanate Surface for Enhanced Cell Adhesion

Junyang Li, Lei Fan, Yanfang Li, Tanyong Wei, Cheng Wang, Feng Li, Hua Tian and Dong Sun

This file includes:

Supporting Figures S1-S4 and Discussions
Supporting Videos S1-S3 Captions

Supplemental Figures S1-S4 and Discussions

The adhesion experiments on microrobots loaded with HEK-293T cells were performed in a microfluidic chip consisted of eight blood vessel–like microchannels to mimic the vascular network. Fig. S1a shows two channels and the microrobots loaded with HEK-293T cells were injected into the inlet of the chip during the experiment. Then, a negative pressure was supplied at the outlet by using a syringe pump to obtain different fluid rate for exerting a fluid impact on the microrobots. In Fig. S1b, an array of cell migration channels was designed to mimic the endothelial layer and cell releasing experiment were conducted in it.

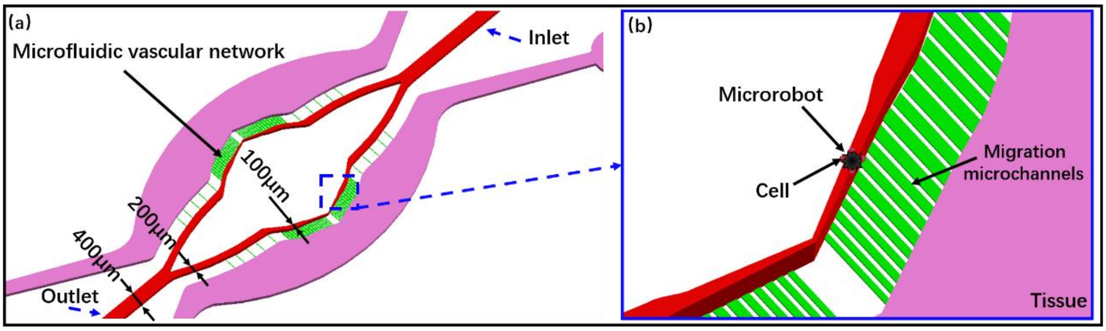

Figure S1. Design of a microfluidic chip.

Volume flow rate/ velocity conversion:

We use the volume flow rate/ velocity conversion formula to calculate the flow velocity:

$$V = Q/S$$

where V represents the flow velocity in the channel of microfluidic chip, S is the cross-sectional area of the microchannel, and Q represents the volume flow rate at a given time. In our experiments, the maximum volume flow rate Q at the inlet is 250μL/min (Approximately equal to 4.1×10^{-9} m^3/s). The diameter in one single microchannel is about 90μm, thus cross-sectional area S is 6.3×10^{-9} m^2. In summary, due to our microfluidic chip has eight microchannels, the flow velocity V in single microchannel is about 8 cm/s.

Force analysis at different flow velocity:

When Re ≪ 1, for spherical microrobot, after it enters the microfluidic channel and stops there, the total force on the microrobot by the fluid can be calculated using the Stokes' law [1]:

$$\boldsymbol{F} = 6\pi\mu R\boldsymbol{v_o}$$

where $\boldsymbol{F}$ is the frictional force, μ is the dynamic viscosity of the fluid, R is the radius of the robot, and $\boldsymbol{v_o}$ is the velocity of the flow. It can be easily found that the velocity and frictional force are positively correlated. In this way, the shear force as a force component tangential to the cross

section also increases with the velocity. As the flow velocity continues to increase, the shear force on the cells loaded on the microrobot becomes greater. Thus, different flow velocity can be used to test the improved adhesion of the designed microrobots.

[1] Li, J.; Li, X.; Luo, T.; Wang, R.; Liu, C.; Chen, S.; Li, D.; Yue, J.; Cheng, S.; Sun, D. Development of a Magnetic Microrobot for Carrying and Delivering Targeted Cells. *Science Robotics* **2018**, *3*.

Fig. S2a shown that a home-designed magnetic manipulation system consists of fixed DT4E-core identical electromagnetic coils with magnetic gradient of 20 T/m. A proportional-integral-derivative controller was used to determine current input to magnetic coils in microrobot motion control. Fig. S2b and Movie S3. show that microrobot moved from initial position to end position in following planned path in a microfluidic chip. Actual and reference trajectories of microrobot in Fig. S2c indicates that the magnetic microrobot can achieve precise navigation to targeted position.

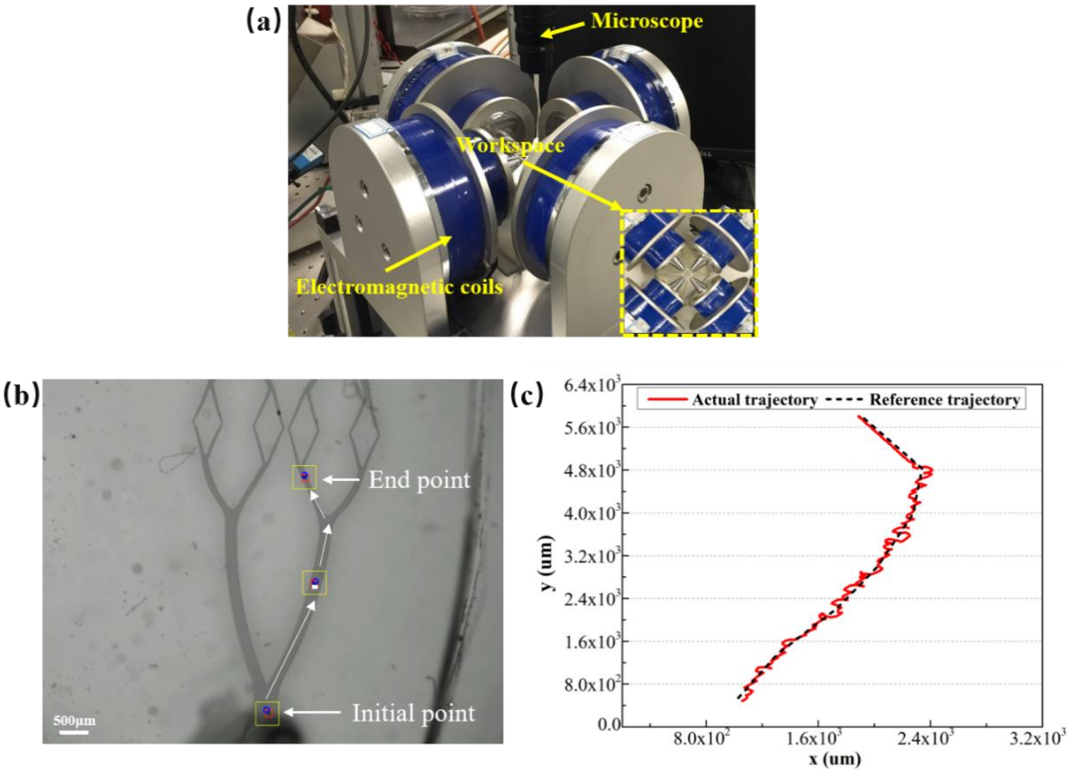

Figure S2. Control of a MSCs-cultured microrobot with NTS in a microfluidic chip. (a) Prototype of the magnetically actuated micromanipulation system. (b) Time-lapsed image of the MSCs-cultured microrobot with NTS moving from initial point to end point. (c) Actual and reference trajectories of microrobot.

The qualitative experiment of protein adsorption in Fig. S3 was proceeded on the glass sample. More protein was adsorbed onto the glass with NTS, suggesting that nanoscale surface shows a higher level of adhesive protein adsorption.

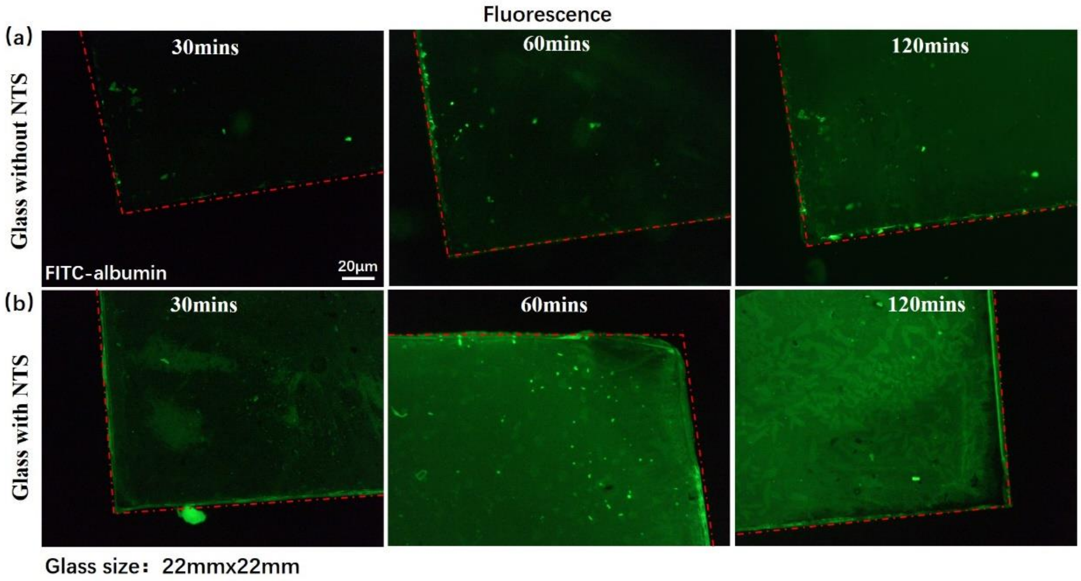

Figure S3. Fluorescence images of the FITC-albumin absorption. (a) Glass without NTS after 30, 60 and 120 minutes incubation. (b) Glass with NTS after 30, 60 and 120 minutes incubation.

Experiments were performed involving the release of HEK-293T cells from the microrobot with NTS in a microfluidic chip (Fig. S4). The cell-cultured microrobot was injected into the inlet of the microfluidic chip. After the microrobots carrying cells arrived in the targeted area, the cells were released from the microrobots and passed through the mimicked endothelial layer. The results proved that the designed microrobot with NTS can firmly anchor cells to avoid fluid impact during the transportation while the cell releasing ability after arriving at the desired site would not be affected.

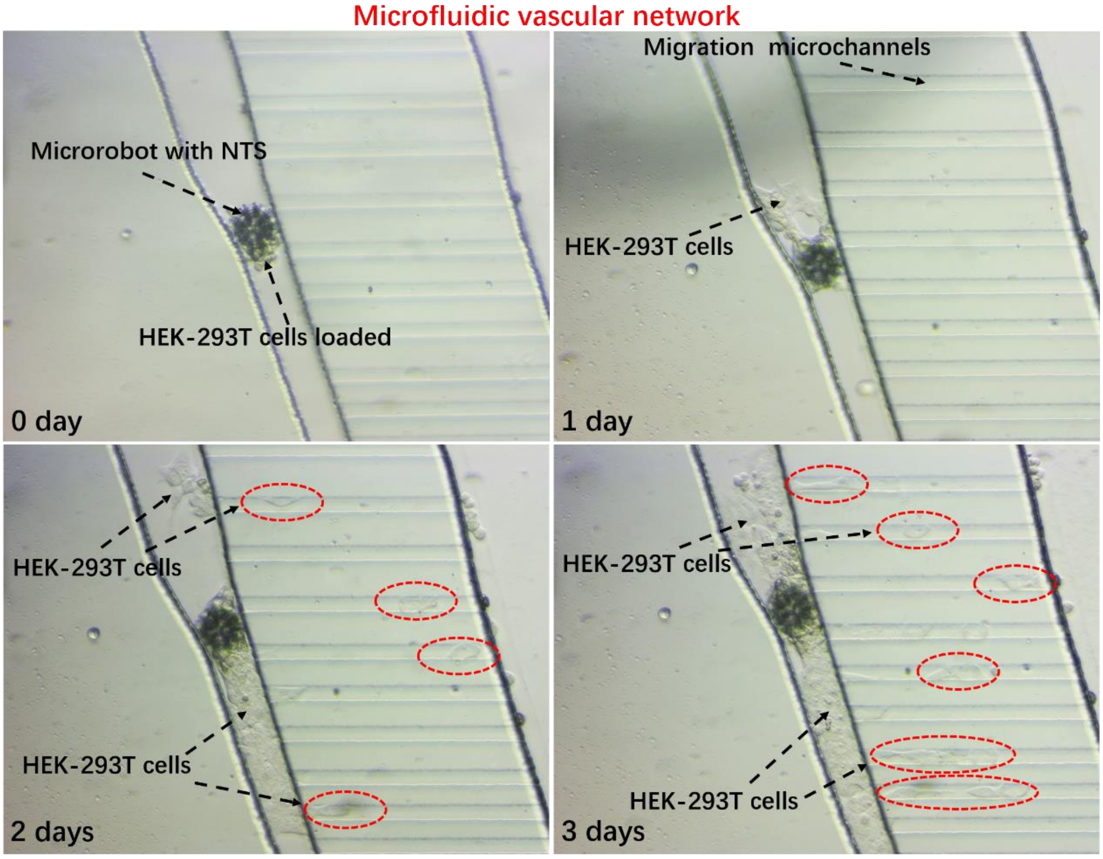

Figure S4. Results of cells releasing ability. The transendothelial migration of the cells released from the microrobots are marked by the red dashed lines.

Supporting Videos S1-S3 Captions

Video S1. Cell adhesion ability of microrobot without NTS in a microfluidic chip.
Video S2. Cell adhesion ability of microrobot with NTS in a microfluidic chip.
Video S3. Magnetic control experiment in a microfluidic chip.
